# Unveiling the Molecular Mechanisms Driving the Capsaicin-Induced Immunomodulatory Effects on PD-L1 Expression in Bladder and Renal Cancer Cell Lines

**DOI:** 10.3390/cancers14112644

**Published:** 2022-05-26

**Authors:** Maria Beatrice Morelli, Oliviero Marinelli, Cristina Aguzzi, Laura Zeppa, Massimo Nabissi, Consuelo Amantini, Daniele Tomassoni, Federica Maggi, Matteo Santoni, Giorgio Santoni

**Affiliations:** 1 School of Pharmacy, University of Camerino, 62032 Camerino, Italy; mariabeatrice.morelli@unicam.it (M.B.M.); oliviero.marinelli@unicam.it (O.M.); cristina.aguzzi@unicam.it (C.A.); laura.zeppa@unicam.it (L.Z.); massimo.nabissi@unicam.it (M.N.); 2 School of Biosciences and Veterinary Medicine, University of Camerino, 62032 Camerino, Italy; consuelo.amantini@unicam.it (C.A.); daniele.tomassoni@unicam.it (D.T.); federica.maggi@unicam.it (F.M.); 3 Medical Oncology Unit, Hospital of Macerata, 62100 Macerata, Italy; mattymo@alice.it

**Keywords:** PD-L1, genitourinary cancer, bladder cancer, renal cell carcinoma, capsaicin, immunotherapy

## Abstract

**Simple Summary:**

Over time, capsaicin (CPS) has been considered both a potential anti-cancer and pro-cancer molecule. Hence, the diversity of CPS functioning has already been established. Now, exploration of its application with immunotherapies might open up a new avenue in cancer therapy. Herein, the application of CPS as an immunoadjuvant to overcome the tumor’s immune-escaping mechanisms or to increase immune checkpoint therapy has been approached. In bladder cancer, the interaction of CPS with its receptor TRPV1 increases PD-L1 expression, promoting a tumorigenic effect and also providing a target for anti-PD-1/PD-L1 immunotherapy. On the contrary, in renal cell carcinoma, CPS downregulates PD-L1 expression in a TRPV1-independent manner, suggesting a potential application of CPS as an immune-adjuvant in this type of cancer.

**Abstract:**

The blockade of the PD-L1/PD-1 immune checkpoint has promising efficacy in cancer treatment. However, few patients with bladder cancer (BC) or renal cell carcinoma (RCC) respond to this approach. Thus, it is important to implement a strategy to stimulate the immune anti-tumor response. In this scenario, our study evaluated the effects of a low capsaicin (CPS) dose in BC and RCC cell lines. Western blot, qRT-PCR and confocal microscopy were used to assess PD-L1 mRNA and protein expression. Alterations to the cellular oxidative status and changes to the antioxidant NME4 levels, mRNA modulation of cytokines, growth factors, transcriptional factors and oncogene, and the activation of Stat1/Stat3 pathways were examined using Western blot, cytofluorimetry and qRT-PCR profiling assays. In BC, CPS triggers an altered stress oxidative-mediated DNA double-strand break response and increases the PD-L1 expression. On the contrary, in RCC, CPS, by stimulating an efficient DNA damage repair response, thus triggering protein carbonylation, reduces the PD-L1 expression. Overall, our results show that CPS mediates a multi-faceted approach. In modulating PD-L1 expression, there is a rationale for CPS exploitation as a stimulus that increases BC cells’ response to immunotherapy or as an immune adjuvant to improve the efficacy of the conventional therapy in RCC patients.

## 1. Introduction

Cancers of the genitourinary (GU) tract are a major source of cancer morbidity and mortality. GU malignancies include bladder and renal cell carcinoma (BC and RCC), the sixth and eighth most common cancers among men and women [1]. BC patients are diagnosed at an early stage and with localized disease, with approximately 70–80% having non-invasive and the remaining 20–30% muscle-invasive disease [2]. RCC, which originates from the renal epithelium, is the most common form of renal cancer (RC) and accounts for >90% of cancers in the kidneys [3]. The disease encompasses several subtypes, of which clear-cell RCC (ccRCC) is the most prevalent and contributes to the most cancer-related deaths [4]. Partial or radical nephrectomy is one of the effective methods to treat ccRCC; but 20–30% of patients treated with surgery will relapse, despite having no evidence of metastases when diagnosed with ccRCC [5]. 

Capsaicin (CPS) is the pungent component of red peppers (*Capsicum frutescens* L.) [6]. Chemically, it is a derivative of vanillyl amide (8-methylN-vanillyl-6-nonenamide). CPS has been found to induce both carcinogenic and anti-carcinogenic effects in a transient receptor potential vanilloid subtype 1 (TRPV1)-dependent and -independent manner [7,8,9,10,11]. In BC cell lines, TRPV1 triggered by CPS induces CD95-mediated apoptosis in an ATM-dependent manner [12]. CPS at high doses reduces proliferation of RCCs in a TRPV1-dependent manner, induces caspase-dependent apoptosis and growth of 786-O RC xenografts in vivo [13]. In BC, CPS triggers the proteasomal degradation of tNOX, leading to the inhibition of NAD+-dependent SIRT1 deacetylase and the enhancement of c-Myc and p53 that suppresses G1 cyclin-dependent kinase activation and triggers cell cycle arrest [14]. Moreover, by downregulating SIRT1, CPS enhances the acetylation of cortactin and β-catenin to decrease MMP-2 and MMP-9 activation and impair cell migration in BC cells [15,16]. CPS suppresses cell proliferation, and induces cell cycle arrest and reactive oxygen species (ROS) production in BC cells, through FOXO3a-mediated pathways [17,18]. CPS also triggers autophagic cell survival, which drives epithelial-mesenchymal transition and chemoresistance in BC cells in a Hedgehog-dependent manner [19]. 

CPS, other than exerting cytotoxic activity in cancer cells, shows immunomodulatory effects, suggesting its potential application in immunotherapy [20]. The identification of TRPV1 in distinct immune cells (e.g., DCs, macrophages and T cells) has opened a new window to the in vitro and in vivo immunomodulatory activity of CPS [21,22]. Dietary CPS in Balb/c mice enhances lymphocyte proliferation and serum IgG levels [23]. CPS stimulates intratumoral anti-cancer immunity by eliciting a T cell-mediated response, leading to the regression of advanced solid tumors [20]. Engagement of TRPV1 on immature DCs by CPS leads to upregulation of antigen-presenting and costimulatory molecules and the maturation of DCs [20]. CPS induces specific damage-associated molecular patterns (DAMPs) immunogenic cell death in human BCs, leading to sensitization of the surrounding stroma and its destruction by tumor-specific T cells [24]. Furthermore, CPS depletes CD4 + CD25 + FoxP3 Treg cells at the tumor site and modulates the cytokine microenvironment (IL-6, IL-12 and GM-CSF increases and IL-10 reduction) [25]. 

In recent years, immunotherapy has substantially improved the therapeutic strategies for the treatment of malignancies. Immune checkpoint inhibitors (ICIs), a class of immunotherapeutic agents that blocks immune inhibitory receptors, or immune checkpoints such as CTLA-4, PD-1 and PD-L1, can reactivate anti-cancer immunity, facilitating tumor elimination [26]. PD-L1 (CD274, B7-H1) is frequently overexpressed in tumors. By binding to PD-1, CPS may inhibit the activation of T lymphocytes to evade the host immune response, preventing tumors from cytotoxic T-lymphocyte-induced killing, and PD-L1 also interacts with B7.1 to further suppress the tumor antigen-induced activation of cytotoxic T lymphocytes [27].

BCs and RCCs have been characterized as tumor groups in which the immunological response is well conserved. PD-L1 is expressed in BCs and RCCs. Recently, studies suggested that it represents a mediator of the stage progression in BC [28], with BC patients expressing high PD-L1 levels showing a poor prognosis and relapse-free survival [29], and with an unfavorable prognosis in immune checkpoint-treated naive ccRCCs [30]. PD-L1, as monotherapy or in combination with other agents, has achieved profound and durable responses in many patients and has been approved by the FDA for use in RCCs and BCs. The blockade of this pathway using specific inhibitors such as pembrolizumab and nivolumab, alone or in combination with ipilimumab and aterizumab, could enhance the cytotoxicity of T cells in the tumor environment and substantially increase the long-term survival in different cancers [26,31,32]. However, although targeting PD-1/PD-L1 has achieved durable responses and disease remission in patients with certain cancers, relatively low response rates and emerging resistance limit its clinical application. Thus, there is a need to further study the molecular mechanisms and direct and indirect drivers regulating PD-L1 expression in cancer cells.

This work aimed to evaluate the effect of a low CPS dose on the PD-L1 mRNA and protein expression in BC and RCC cell lines, along with the molecular mechanisms behind CPS-mediated immunomodulatory effects. 

## 2. Materials and Methods

### 2.1. Cell Lines

T24 and 5637 BC and A498 RCC lines were sourced from American Type Culture Collection (Rockville, MD, USA). T24 and 5637 BC were maintained in RPMI-1640 medium (Euroclone, Milan, Italy) supplemented with 10% heat-inactivated fetal calf serum (Euroclone), 2 mM L-glutamine, 100 IU/mL penicillin and 100 μg/mL streptomycin. A498 were maintained in MEM medium (Euroclone) supplemented with 10% heat-inactivated fetal calf serum, 2 mM L-glutamine, 100 IU/mL penicillin and 100 μg/mL streptomycin. All cell lines were maintained at 37 °C with 5% CO_2_ and 95% humidity.

### 2.2. Reagents

CPS ([N-(4-hydroxy-3-methoxy-phenyl)methyl]-8-methyl-6-nonenamide), CPZ (N- [2-(4-chlorophenyl)ethyl]-1,3,4,5-tetrahydro-7,8-dihydroxy-2H-2-benzazepine-2-carbothioamide), N-acetyl L-cysteine (NAC), 3-(4,5-dimethylthiazol2-yl)-2,5-diphenyltetrazolium bromide (MTT), dichlorodihydrofluoresceindiacetate (DCFDA) and carbonyl cyanide chlorophenylhydrazone (CCCP) were obtained from Sigma–Aldrich (Milan, Italy). Propidium iodide (PI), Annexin V-FITC and 5,5′,6,6′-tetrachloro-1,1′,3,3′-tetraethylbenzimidazolcarbocyanine iodide (JC-1) were obtained from Invitrogen (Milan, Italy). CPS and CPZ stock solutions were dissolved in dimethyl sulfoxide.

### 2.3. MTT Assay

T24 and 5637 BC and A498 RCC cells (8 × 10^4^/mL) were plated in 96-well plates and treated for 24, 48 and 72 h with different doses of CPS (1–500 μM). At the end of the treatment, 0.8 mg/mL MTT was added to the samples and incubated for 3 h. The medium was removed from the wells, the formazan crystals were dissolved with 100 µL/well DMSO and the absorbance was read by a microtiter plate spectrophotometer (BioTek Instruments, Winooski, VT, USA). Four replicates were carried out for each treatment.

### 2.4. ROS Analysis

DCFDA was used to quantify the number of reactive oxygen species (ROS) present within cells. Cells were treated for up to 24 h with vehicle or CPS 50 μM, alone or pretreated with NAC (10 mM), and stained with 20 μM DCFDA for 45 min at 37 °C and 5% CO_2_. The intensity of the fluorescence was analyzed on a FACScan cytofluorimeter using CellQuest software.

### 2.5. Mitochondrial Transmembrane Potential (∆Ψm) Assay

ΔΨ_m_ was evaluated by JC-1 staining. Briefly, 2 × 10^5^ cells treated with 50 μM CPS for different lengths of time were incubated for 10 min at room temperature with 10 μg/mL JC-1 and then analyzed by a FACScan cytofluorimeter. Green (530 nm) and red (570 nm) emission fluorescence scores were collected simultaneously. Data were analyzed using Cell Quest software version 5.1. CCCP, a mitochondrial uncoupler that collapses ΔΨ_m_, was used as a positive control.

### 2.6. Annexin V and PI Staining

Cell death was evaluated using Annexin V-FITC and PI staining. To that end, 5637, T24 and A498 cells (3 × 10^5^/mL) were treated with CPS or with the vehicle for up to 24 h, then incubated with 5 μL Annexin V-FITC or 20 μg/mL PI for 10 min at room temperature. The percentage of positive cells determined over 10,000 events was analyzed on a FACScan cytofluorimeter using CellQuest software. 

### 2.7. Western Blot Analysis

Cells were lysed in lysis buffer (1 M Tris pH 7.4, 1 M NaCl, 10 mM EGTA, 100 mM NaF, 100 mM NaVO_4_, 100 mM phenylmethylsulfonyl fluoride, 2% deoxycholate, 100 mM EDTA, 10% Triton X-l00, 10% glycerol, 10% SDS, 0.1 M Na_4_P_2_O_7_) containing a protease inhibitor cocktail (Sigma-Aldrich). Lysates were separated on SDS polyacrylamide gel and transferred onto Hybond-C extra membranes (GE Healthcare, Milan, Italy). Membranes were incubated overnight at 4 °C in primary Abs (anti-H2AX 1:1000, Cell Signaling, Milan, Italy; anti-PD-L1 1:300, Santa Cruz Biotechnology, Milan, Italy; anti-TRPV1, 1:1000, Invitrogen; anti-GAPDH, 1:1000, Santa Cruz; anti-NME4, 1:2000, GeneTex, Irvine, CA, USA; anti-Stat1, 1:1000, Cell Signaling, anti-Stat3, 1:2000, Cell Signaling; anti-phospho-Stat1 T701, 1:1000, Cell Signaling; anti-phospho-Stat3 S727, 1:1000, Cell Signaling; anti-phospho-Stat3 Y705, 1:1000, Cell Signaling; anti-ATM (phospho S1981), 1:1000, Abcam, Cambridge, UK; anti-ATM, 1:1000, Cell Signaling; anti-CHK2, 1:1000, Cell Signaling; anti-phospho-CHK2 (Ser19), 1:1000, Cell Signaling; anti-phospho-CHK2 (Ser33–35), 1:1000, Cell Signaling; anti-phospho-CHK2 (Ser516), 1:1000, Cell Signaling; anti-phospho-CHK2 (Thr68) (C13C1), 1:1000, Cell Signaling; anti-β-actin, 1:1000, Santa Cruz Biotechnology), followed by incubation at room temperature for 1 h with HRP-conjugated anti-mouse or anti-rabbit secondary Abs (Cell Signaling). Peroxidase activity was visualized with the LiteAblot ^®^PLUS (Euroclone) kit and densitometric analysis was carried out by a Chemidoc using Quantity One software (BioRad, Milan, Italy). For quantification, GAPDH or β-actin was used as the loading control. One representative of three independent experiments is shown.

### 2.8. RT Profiler PCR Array

The total RNA from T24, 5637 and A498 cells, untreated or treated for 4 or 12 h with CPS (50 μM), was isolated as described above. Two micrograms of RNA extracted from each sample were retrotranscribed in a total volume of 20 μL using Reaction Ready^TM^ first-strand cDNA (Superarray Bioscience Corporation, Frederick, MD, USA). Quantitative RT-PCR was performed using an IQ5 Multicolor Real-Time PCR Detection System (BioRad), Super Array’s RT2 Real-Time SYBR Green PCR Master Mix and the Human Pathways Cancer Finder^TM^ and Human Apoptosis plates (Superarray Bioscience Corporation). Each PCR amplification consisted of heat activation for 10 min at 95 °C, followed by 40 cycles of 95 °C for 15 s and 60 °C for 1 min. Measurement of five housekeeping genes’ levels on the samples was used to normalize the mRNA content, and the expression levels of 168 different genes were expressed as the relative fold of the corresponding control according to the protocol.

### 2.9. Confocal Laser Scanning Microscopy Analysis

A498, 5637 and T24 cells were grown on 18 × 18 polylysine-coated slides in a fresh medium. After 72 h, cells were treated with CPS 50 μM for 12 and 24 h, then fixed with 2% and 4% paraformaldehyde, with 0.3% Triton X-100 in PBS, for 10 min at room temperature. The cells were washed in PBS and incubated for 1 h at room temperature with 3% bovine serum albumin (BSA) and then overnight at 4 °C with anti-PD-L1 Ab diluted 1:500 in 0.3% Triton X-100 in PBS. Slides were washed with PBS and incubated for 30 min at 37 °C with anti-mouse Alexa Fluor-594 goat (Thermo Fisher Scientific, Rome, Italy) diluted 1:100. After washing in PBS, slides were incubated with DAPI diluted 1:200 in 0.3% Triton X-100 in PBS for 45 min. To assess the immunostaining background, a group of slides was incubated with a non-immune serum instead of the primary antibody. Sections were analyzed using a Nikon mod. C2 plus Confocal Laser Microscope (Nikon Corporation, Minato City, Japan), and the densitometric analysis was performed by NIS Elements Nikon image analyzer software (Nikon, Florence, Italy) to analyze the mean intensity of immunofluorescence (MFI).

### 2.10. Oxidized Protein Analysis

An OxyBlot Protein Oxidation Detection Kit (Merck Millipore, Burlington, MA, USA) was used to detect the oxidized proteins, according to the manufacturer’s instructions. Dinitrophenyl hydrazine was added to the crude total proteins (20 μg) to derive the carbonyl groups from the protein side-chains. Carbonylated proteins were resolved using SDS-polyacrylamide gel electrophoresis, and the Western blot analysis was performed using the provided anti-DNP antibody (1:150). Detection was carried out using LiteAblot PLUS or Turbo Kits (Euroclone), and DNP signals were quantified by densitometry using Quantity One software (BioRad). 

### 2.11. TRPV1 Silencing

siTRPV1 and siGLO nontargeting (used as control) FlexiTube siRNA were sourced from Qiagen (Milan, Italy). T24 and 5637 cells were plated at 2.5 × 10^4^/mL and siTRPML1 or siGLO (150 ng) was added to the wells, following the HiPerfect Transfection Reagent protocol (Qiagen). Cells were harvested at 72 h post-transfection. Silencing efficiency was evaluated by qRT-PCR and Western blot. No differences were observed when comparing siGLO transfected with untransfected cells.

### 2.12. Statistical Analysis

The statistical significance was determined by Student’s *t*-test and one-way ANOVA.

## 3. Results

### 3.1. CPS Induces DNA Damage in Living 5637, T24 and A498 Cancer Cell Lines

**Figure 1:**
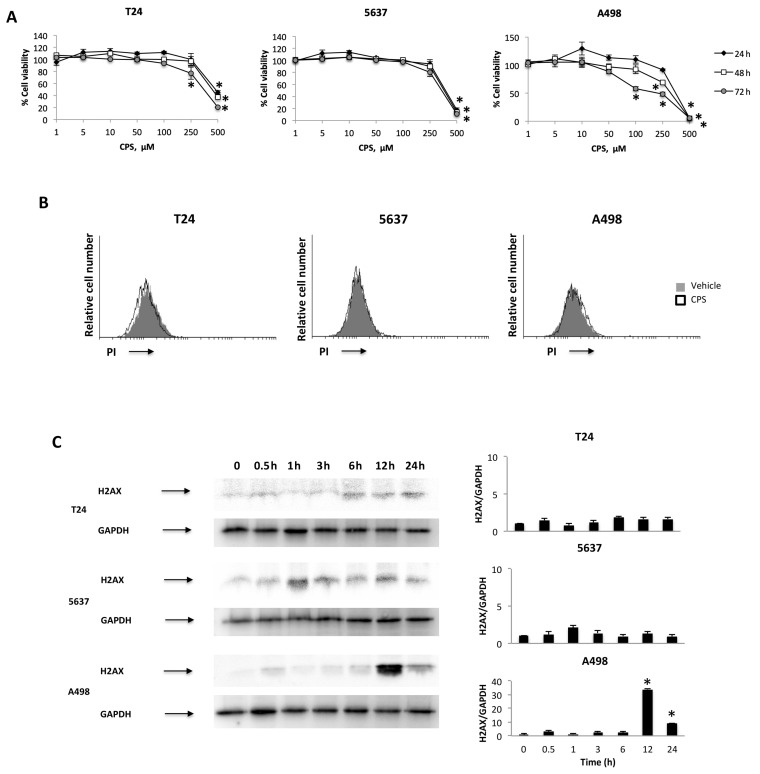
CPS effects on T24, 5637 and A498 cell lines. (**A**) Cell viability was evaluated by 3-(4,5-dimethylthiazol-2-yl)-2,5-diphenyltetrazolium bromide (MTT) assay in T24, 5637 and A498 cells treated with different doses of CPS for up to 72 h. Data shown are expressed as mean ± SE of three separate experiments; * *p* < 0.05. (**B**) T24, 5637 and A498 cells were treated with 50 μM CPS for 24 h and then PI incorporation was analyzed by flow cytometry. Histograms are representative of one of three separate experiments. (**C**) Representative immunoblots for H2AX expression levels in total cellular lysates from T24, 5637 and A498 cells treated with CPS for up to 24 h. Blots are representative of one of three separate experiments. H2AX densitometry values were normalized to GAPDH, used as the loading control. The H2AX protein levels were determined with respect to time 0. Data are expressed as mean ± SD; * *p* < 0.001. Detailed information about the Western blotting can be found in Figure S5.

We initially evaluated the effects of different doses (1 to 500 μM) of CPS at different times (24, 48 and 72 h) on the growth of T24, 5637 and A498 cells by MTT assay. We found that CPS in a low dose range (1–50 μM) does not affect the viability of BC and RCC cells (Figure 1A). At higher doses (250–500 μM), CPS reduces—mainly at 72 h—the growth of T24, 5637 and A498 cells. To confirm these data, we stained CPS-treated T24, 5637 and A498 cells with PI, and the percentage of dead cells was evaluated by cytofluorimetric analysis. No PI-positive cells were observed in 50 μM CPS-treated T24, 5637 and A498 cells up to 24 h (Figure 1B). To further evaluate possible DNA damage induced by a non-toxic CPS dose, we analyzed the presence of γ-H2AX (H2AX), a phosphorylated variant of histone 2A that is associated with DNA double-strand breaks (DSB) [33] in T24, 5637 and A498 cells at different times (0.5–24 h) after CPS treatment. Interestingly, Western blot analysis revealed a different response of BC cells, versus RCC cells, to CPS exposure. Low H2AX expression was evidenced just at 1 h and 6 h in T24 and 5637 cells, respectively, compared to untreated cells, whereas high H2AX levels were evidenced at 12–24 h after CPS treatment in A498 cells (Figure 1C). Moreover, no Annexin V-positive apoptotic cells were observed by cytofluorimetric and FACS analysis in 50 μM CPS-treated cells monitored for up to 24 h (Figure S1). These results suggest that a low CPS dose induces DNA damage in PI-negative living cancer cells.

### 3.2. CPS-Induced DNA Damage Modulates the PD-L1 mRNA and Protein Expression in T24, 5637 and A498 Cells

**Figure 2:**
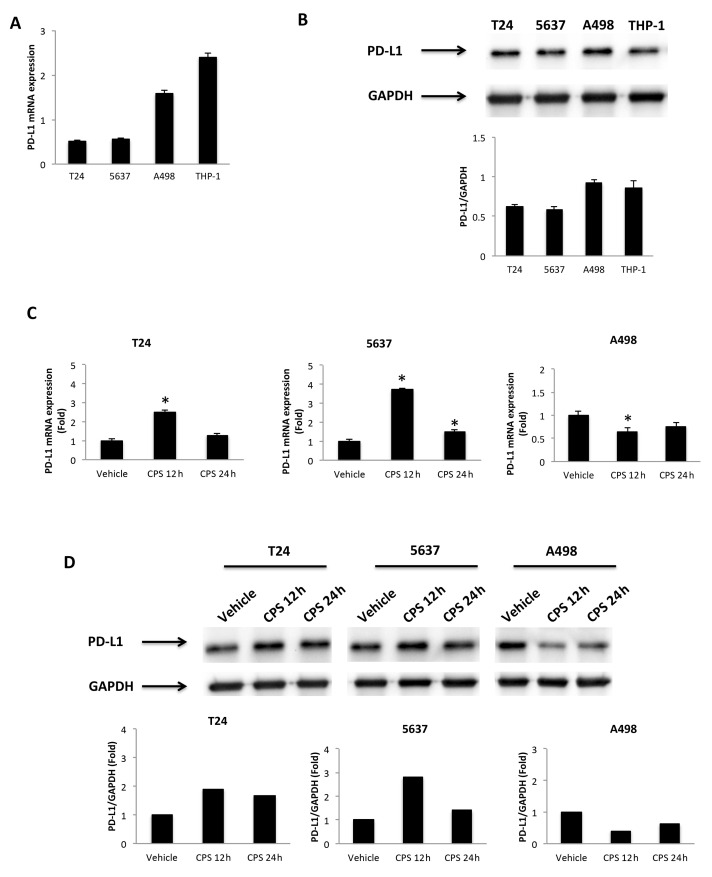
CPS treatment influences PD-L1 expression. (**A**) The relative PD-L1 mRNA expression in T24, 5637, A498 and THP-1 (positive control) cell lines was evaluated by qRT-PCR. PD-L1 mRNA levels were normalized for GAPDH expression. Data are expressed as mean ± SD. (**B**) Western blot analysis of PD-L1 protein levels. Blots are representative of one of three separate experiments. PD-L1 densitometry values were normalized to GAPDH, used as the loading control. Data are expressed as mean ± SD. (**C**) PD-L1 mRNA expression in T24, 5637 and A498 cell lines treated with 50 μM CPS for 12 and 24 h was evaluated by qRT-PCR. PD-L1 mRNA levels were normalized for GAPDH expression. Data are expressed as mean ± SD; * *p* < 0.05. (**D**) Western blot analysis of PD-L1 protein expression in T24, 5637 and A498 cell lines treated with 50 μM CPS for 12 and 24 h. Blots and densitometry values are representative of one of three separate experiments. PD-L1 densitometry values were normalized to GAPDH, used as the loading control. The PD-L1 protein levels of treated cells were determined with respect to PD-L1 levels of vehicle-treated cells. Detailed information about the Western blotting can be found in Figure S6.

Firstly, we evaluated the expression of the PD-L1 mRNA and protein levels in T24, 5637 and A498 cells (Figure 2A,B), as well as in the THP-1 cell line used as a positive control [34]. Then, to evaluate the responsiveness to CPS (50 μM), the PD-L1 mRNA and protein levels were evaluated at 12 and 24 h after treatment in T24, 5637 and A498 cells. We found that CPS increased the PD-L1 expression, both at mRNA and protein levels, in T24 and 5637 cells compared to vehicle-treated cells; on the contrary, a marked reduction in PD-L1 mRNA and protein expression was evidenced in A498 cells at 12 and 24 h after CPS treatment (Figure 2C,D). No major differences were observed when comparing untreated vs. vehicle-treated cells.

### 3.3. Distribution of PD-L1 Protein in CPS-Treated T24, 5637 and A498 Cells

**Figure 3:**
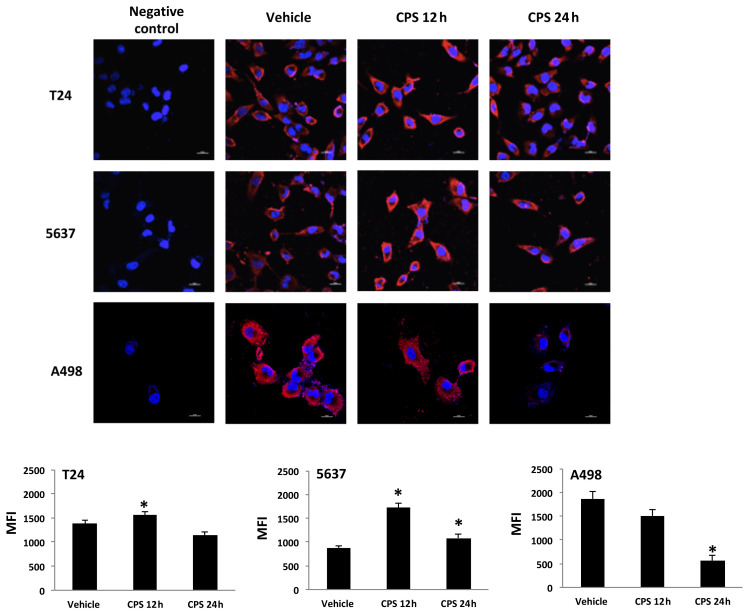
PD-L1 localization in CPS-treated cells. Confocal microscopy analysis of PD-L1 expression in T24, 5637 and A498 cells treated with CPS. Cells were stained with anti-human PD-L1 followed by Alexa Fluor-594 secondary Ab. We used 40,6-diamidino-2-phenylindole (DAPI) to counterstain nuclei. MFI = mean fluorescence intensity. Data are expressed as mean ± SEM; * *p* < 0.01 vs. vehicle-treated cells.

It has been demonstrated that PD-L1 protein has multiple subcellular localizations. Thus, we evaluated its specific siting in CPS-treated T24, 5637 and A498 cells by confocal microscopy (Figure 3). We found that PD-L1 protein localizes in the plasma membrane and cytoplasm. Moreover, CPS treatment significantly increased PD-L1 levels in T24 and 5637 cells at 12 h after CPS treatment. On the contrary, reduced PD-L1 protein expression was evidenced in A498 cells mainly at 24 h after CPS treatment (Figure 3).

### 3.4. CPS-Induced DSB DNA Damage Is Associated with p53/ATM/CHK2 and BRCA1/hTERT mRNA Expression in T24, 5637 and A498 Cells

**Figure 4:**
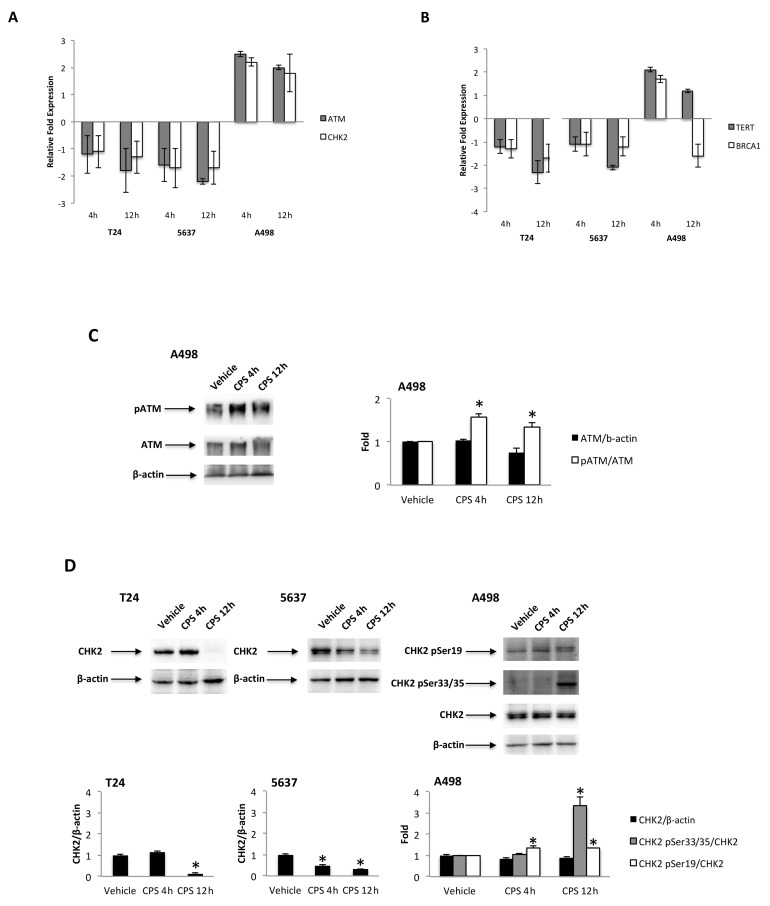
(**A**) Relative ATM and CHK2 mRNA expression in T24, 5637 and A498 cells treated with CPS for 4 and 12 h was evaluated by quantitative reverse transcription (qRT)-PCR. ATM and CHK2 mRNA levels were normalized for GAPDH expression and expressed as the fold with respect to the vehicle. (**B**) Relative TERT and BRCA1 mRNA expression in T24, 5637 and A498 cells treated with CPS for 4 and 12 h was evaluated by qRT-PCR. TERT and BRCA1 mRNA levels were normalized for GAPDH expression. Data are expressed as mean ± SD. (**C**) Immunoblot representative of pATM and ATM protein levels in A498 cell line. pATM densitometry values were normalized to ATM, and ATM densitometry values were normalized for β-actin expression, used as the loading control. Data are expressed as mean ± SD; * *p* < 0.05 vs. vehicle-treated cells. (**D**) Immunoblot representative of total and phosphorylated CHK2 protein levels in T24, 5637 and A498 cell lines. Densitometry values of CHK2 phosphorylated forms were normalized to total CHK2, and CHK2 densitometry values were normalized for β-actin expression, used as the loading control. Data are expressed as mean ± SD; * *p* < 0.05 vs. vehicle-treated cells. Detailed information about the Western blotting can be found in Figure S7.

Tumor suppressor p53 protein regulates the cellular response to DNA damage [35], and activation of p53 by the ATM/CHK2 signaling pathway is critical for p53-dependent transactivation [36,37]. The ATM/CHK2 pathway serves as a DNA damage sensor that promotes DNA repair and cell cycle arrest [36]. Thus, the mRNA expression of the p53/ATM/CHK2 signaling pathway at different times (4 and 12 h) after CPS (50 μM) treatment was evaluated in p53-mutated T24 and 5637 cells and in p53 wild-type A498 cells by the Human Cancer Pathway Finder^TM^ RT2-Profiler^TM^ PCR Array and qRT-PCR. We found that p53 was induced ex-novo at 4 h after CPS treatment, and its expression progressively increased at 12 h in the A498 cells. Then, we determined whether the modulation of PD-L1 expression induced by CPS exposure was associated with changes in the ATM and CHK2 expression. Increased ATM and CHK2 mRNA expression were detected at 4 h after CPS treatment in A498 cells compared to vehicle-treated cells (Figure 4A). On the other hand, significant inhibition of the ATM and CHK2 mRNA levels was observed in CPS-treated T24 and 5637 cells at 12 h after treatment (Figure 4A). The ATM/CHK2 signaling pathway was also assessed at protein levels. Increased ATM phosphorylation levels were observed at 4 h after CPS treatment in A498 cells compared to vehicle-treated cells (Figure 4C). Instead, no ATM Ser1981 phosphorylation and no changes in ATM total levels were observed in CPS-treated T24 and 5637 cells compared to vehicle-treated cells. Results for ATM activation in A498 cells were supported by enhanced Ser19-CHK2 and Ser33/35-CHK2 phosphorylation after CPS treatment (Figure 4D). No significant changes in CHK2 total levels (Figure 4D), nor in Tyr68-CHK2 and Ser516-CHK2 phosphorylation levels, were found in CPS-treated A498 cells. In T24 and 5637 cells, CPS administration led to a decrease in the total CHK2 protein levels (Figure 4D); no changes in the CHK2 phosphorylation status were observed during the observation period.

CPS-induced DNA damage results in genomic instability and defects in DNA repair. Thus, the human telomerase reverse transcriptase (hTERT) and breast cancer 1 (BRCA1) expression levels were evaluated by qRT-PCR in CPS-treated T24, 5637 and A498 cells at 4 and 12 h after treatment. Reduced hTERT and no significant changes in BRCA1 mRNA expression were evidenced in CPS-treated T24 and 5637 cells. On the contrary, enhanced hTERT and BRCA1 mRNA expression were observed at 4 h for CPS-treated A498 cells (Figure 4B).

### 3.5. TRPV1- or ROS-Dependent Signals Induced in CPS-Treated T24, 5637 and A498 Cells

**Figure 5:**
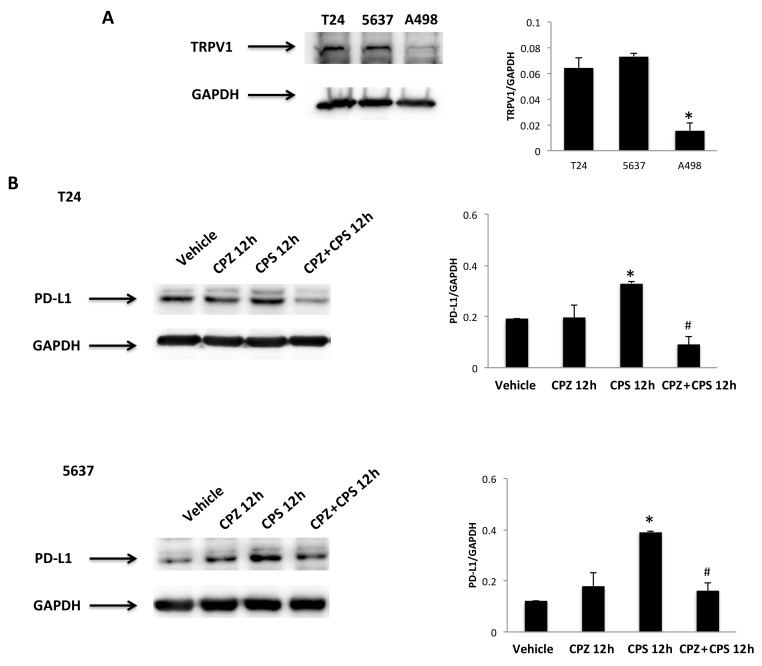
CPS treatment influences PD-L1 expression in a TRPV1-dependent manner. (**A**) Immunoblot representative of TRPV1 expression in T24, 5637 and A498 cell lines. TRPV1 densitometry values were normalized to GAPDH, used as the loading control. Data are expressed as mean ± SD; * *p* < 0.001 vs. BC cells. (**B**) Immunoblot representative of TRPV1 expression in T24 and 5637 cells pretreated with CPZ and then stimulated with CPS for 12 h. TRPV1 densitometry values were normalized to GAPDH, used as the loading control. Data are expressed as mean ± SD; * *p* < 0.01 vs. vehicle-treated cells, ^#^ *p* < 0.05 vs. CPS-treated cells. Detailed information about the Western blotting can be found in Figure S8.

An oxidative state and mild hyperthermic signals can modulate immune checkpoint expression in cancer cells [38]. Given that CPS is a highly selective agonist for the TRPV1 channel [39] and TRPV1 is a heat signal transducer [8], the expression of the TRPV1 protein was evaluated in 5637, T24 and A498 cells by Western blot analysis. As previously reported, a band of 95 kDa, corresponding to the TRPV1 protein, was evidenced in both BC cell lines [40], whereas negligible TRPV1 protein expression was observed in A498 cells [41] (Figure 5A). We assessed the anti-TRPV1 Ab efficacy in 5637 and T24 cell lines by qRT-PCR and Western blot analysis. Both TRPV1 mRNA and protein levels were decreased by about 80% in T24 and 70% in 5637 cells silenced cells after 72 h of transfection, as shown in Figure S2. Then, to evaluate whether the CPS immunomodulatory effect on PD-L1 expression is TRPV1-dependent, the capability of the TRPV1 antagonist, CPZ (5 μM; 10:1 CPS/CPZ ratio), to revert the CPS-induced increase in PD-L1 protein was evaluated in T24 and 5637 cells. As shown in Figure 4B, the CPZ antagonist completely reverts the CPS-induced PD-L1 protein increase in both BC cell lines at 12 h after treatment (Figure 5B).

**Figure 6:**
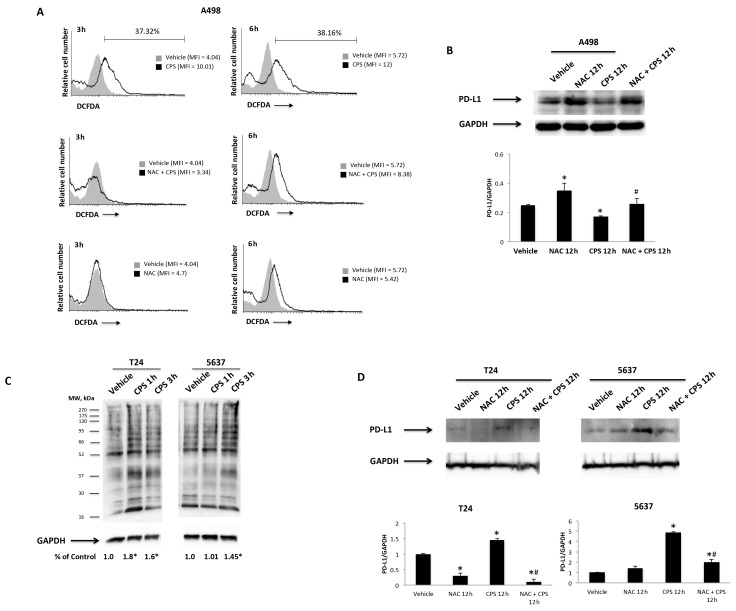
CPS influences PD-L1 protein levels via ROS production. (**A**) ROS generation in A498 cells pretreated with NAC for 1 h and then treated with 50 μM CPS for up to 24 h. Cells were stained with DCFDA before flow cytometric analysis. MFI = mean fluorescence intensity. (**B**) Immunoblot representative of PD-L1 expression in A498 cells pretreated with NAC and then stimulated with CPS for 12 h. PD-L1 densitometry values were normalized to GAPDH, used as the loading control. Data are expressed as the mean ± SD; * *p* < 0.05 vs. vehicle-treated cells, ^#^ *p* < 0.05 vs. CPS treated cells. (**C**) Carbonyl groups generated by oxidative stress were subjected to DNPH derivatization, and increases in oxidatively modified proteins were detected with an antibody against DNP after size fractionation followed by Western blotting. Quantification of total protein carbonylation was performed, normalizing the densitometry values to GAPDH, used as the loading control. The image is representative of three independent experiments; * *p* < 0.01 vs. vehicle-treated cells. (**D**) Immunoblot representative of PD-L1 expression in T24 and 5637 cells pretreated with NAC and then stimulated with CPS for 12 h. PD-L1 densitometry values were normalized to GAPDH, used as the loading control. Data are expressed as the mean ± SD; * *p* < 0.05 vs. vehicle-treated cells, ^#^ *p* < 0.05 vs. CPS treated cells. Detailed information about the Western blotting can be found in Figure S9.

Then, ROS production, as well as the mitochondrial transmembrane potential, were measured by DCFDA and tetraethylbenzimidazolylcarbocyanine iodide (JC-1) staining and cytofluorimetric and FACS analysis in T24, 5637 and A498 cells treated for different times (3, 6, 12 and 24 h) with 50 μM CPS. The results showed that treatment with CPS at 3–6 h induces ROS production in A498 cells (Figure 6A), but not in T24 and 5637 cells at any time after CPS treatment, compared to vehicle-treated cells (Figure S3). Moreover, to confirm the CPS-induced ROS production, we pretreated CPS-treated A498 cells with the ROS inhibitor, NAC, for 1 h. ROS generation was completely reverted by NAC in CPS-treated A498 cells at 3–6 h treatment (Figure 6A). Finally, to correlate CPS-induced ROS generation with CPS-induced PD-L1 downregulation, we also demonstrated by Western blot that NAC restores PD-L1 protein levels in A498 cells at 12 h after treatment (Figure 6B).

Oxidative stress can also be mediated by the presence of carbonyl groups that trigger protein peroxidation [42]. Thus, to evaluate whether CPS treatment can lead to the accumulation of carbonylated proteins, we assessed the presence of this kind of oxidative modification in CPS-treated T24 and 5637 cells, using the OxyBlot^TM^ Protein Oxidation Kit and Western blot analysis, after derivatizing carbonyl groups’ moieties with 2,4-dinitrophenylhydrazine (DNPH) (Figure 6C). This technique was validated by omitting the DNPH treatment, anti-DNP antibody or secondary anti-rabbit IgG antibody. A higher intensity of carbonyl staining was evidenced at 1–3 h in CPS-treated T24 and 5637 cells. Moreover, to confirm the relationship between CPS-induced carbonyl stress and PD-L1 upregulation, we also demonstrated by Western blot analysis that NAC inhibition of carbonylation [43] completely reverts the CPS effects on PD-L1 protein expression in T24 and 5637 cells at 12 h after treatment (Figure 6C). Finally, no changes in mitochondrial transmembrane potential (∆Ψm) were evidenced after CPS treatment in 5637, T24 and A498 cells (Figure S4). Thus, CPS triggers oxidative stress in a TRPV1-dependent manner in T24 and 5637 cells, and in an independent manner in A498 cells.

### 3.6. Transcriptional Factors, Oncogenes, Cytokines and Growth Factors Are Associated Gene-Promoting Drivers in CPS-Induced Modulation of PD-L1 Expression in 5637, T24 and A498 Cells

The gene profile was evaluated by qRT-PCR using the Human Cancer PathwayFinder^TM^ RT2Profiler^TM^ PCR Array in 5637, T24 and A498 cells treated for 4 and 12 h with CPS (50 μM) or vehicle (DMSO) (Table 1 and Table 2). No major differences were evidenced when comparing vehicle-treated vs. untreated T24, 5637 and A498 cells. In this regard, CPS-induced DNA damage enhances the Interferon α1 and β1 (IFNα/β) mRNA expression in T24 and 5637 cells, but not in A498 cells, at 4–12 h after treatment. In addition, enhanced TGFβ1, TGFβR1 and FGFR2 mRNA expression were detected in CPS-treated T24 and 5637 cells. In parallel, in CPS-treated A498 cells, PDGFα induction and TNFα and TNFRS (TNFRS10b, TNFRS1A, TNFRS25) increases were observed at 4–12 h after treatment. Changes in PD-L1 expression induced by the DNA damage response also involve the modulation of the transcriptional activity. So, a marked downregulation of NF-kB, Fos, Jun and Myc transcriptional factors (TFs) was evidenced in CPS-treated T24 and 5637 cells; in CPS-treated A498 cells, enhanced mRNA expression of NF-kB, E2F1 and MYC was evidenced. The DNA damage modulates several oncogenes; here, ERBB2 and MET mRNA expression increased in CPS-treated A498 cells, whereas ETS2, ERBB2 and AKT mRNA expression decreased in T24 and 5637 cells. Finally, SYK and MAPKK1 signaling molecules showed increased expression only in CPS-treated A498 cells.

### 3.7. Nucleoside Diphosphate Kinase (NDPK)/Nm23-H4 Played a Role in the CPS-Induced PD-L1 Protein Expression Modulation in T24 and 5637 Cells

**Figure 7:**
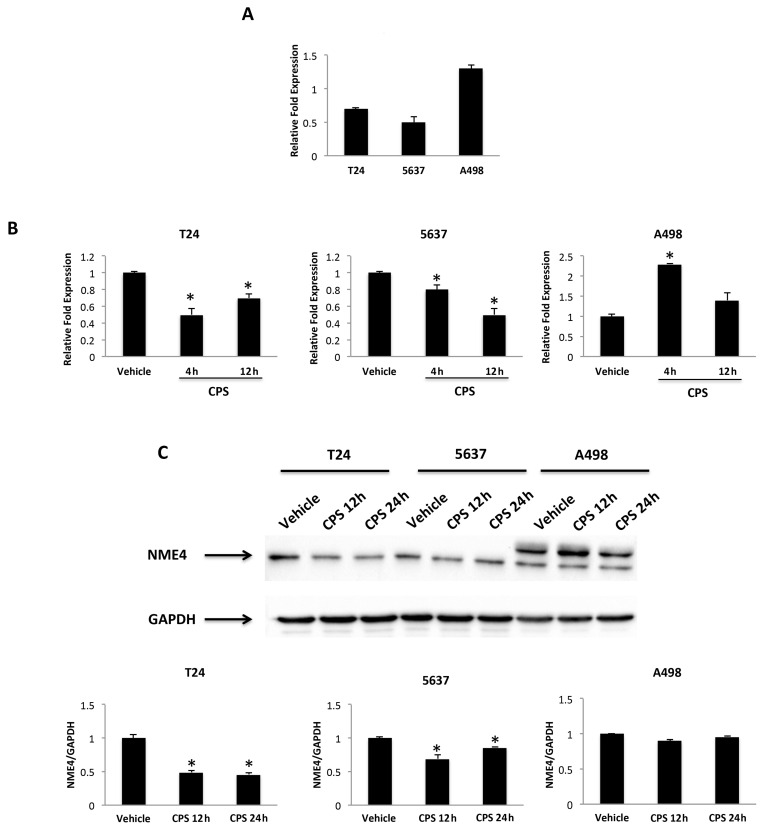
CPS treatment influences NME4 expression. (**A**) Relative NME4 mRNA expression in T24, 5637 and A498 cell lines was evaluated by qRT-PCR. NME4 mRNA levels were normalized for GAPDH expression. Data are expressed as mean ± SD. (**B**) Relative NME4 mRNA expression in T24, 5637 and A498 cell lines treated with CPS for 4 and 12 h was evaluated by qRT-PCR. NME4 mRNA levels were normalized for GAPDH expression. Data are expressed as mean ± SD; * *p* < 0.05 vs. vehicle-treated cells. (**C**) Western blot analysis of NME4 protein levels in T24, 5637 and A498 cell lines treated with CPS for 12 and 24 h. Blots are representative of one of three separate experiments. NME4 densitometry values were normalized to GAPDH, used as the loading control. Data are expressed as mean ± SD; * *p* < 0.05 vs. vehicle-treated cells. Detailed information about the Western blotting can be found in Figure S10.

Oxidative stress and the type I interferon signaling pathway [44] regulate the expression and function of the anti-oxidative nucleoside diphosphate kinase (NDPK)/Nm23-H4 protein. Thus, we evaluated the capability of 50 μM CPS to modulate NME4 expression. Via qRT-PCR, reduced NME4 mRNA levels were observed in CPS-treated T24 and 5637 cells at 12–24 h after treatment; on the contrary, a marked enhancement of NME4 mRNA levels, both at 4 and 12 h, was evidenced in CPS-treated A498 cells. To confirm NME4 modulation, protein expression levels were also analyzed. Given that the product encoded by NME4 targets mitochondria via an N-terminal-specific sequence, which is cleaved to reveal catalytic activity [45], we evaluated the NME4 active form. In particular, CPS treatment reduces at 12–24 h the NME4 protein levels in T24 and 5637, whereas no modulation of the active form is evidenced in A498 cells compared to vehicle-treated cells (Figure 7A,B).

### 3.8. CPS-Induced PD-L1 Modulation Requires Stat1/Stat3 Activation in 5637, T24 and A498 Cells

**Figure 8:**
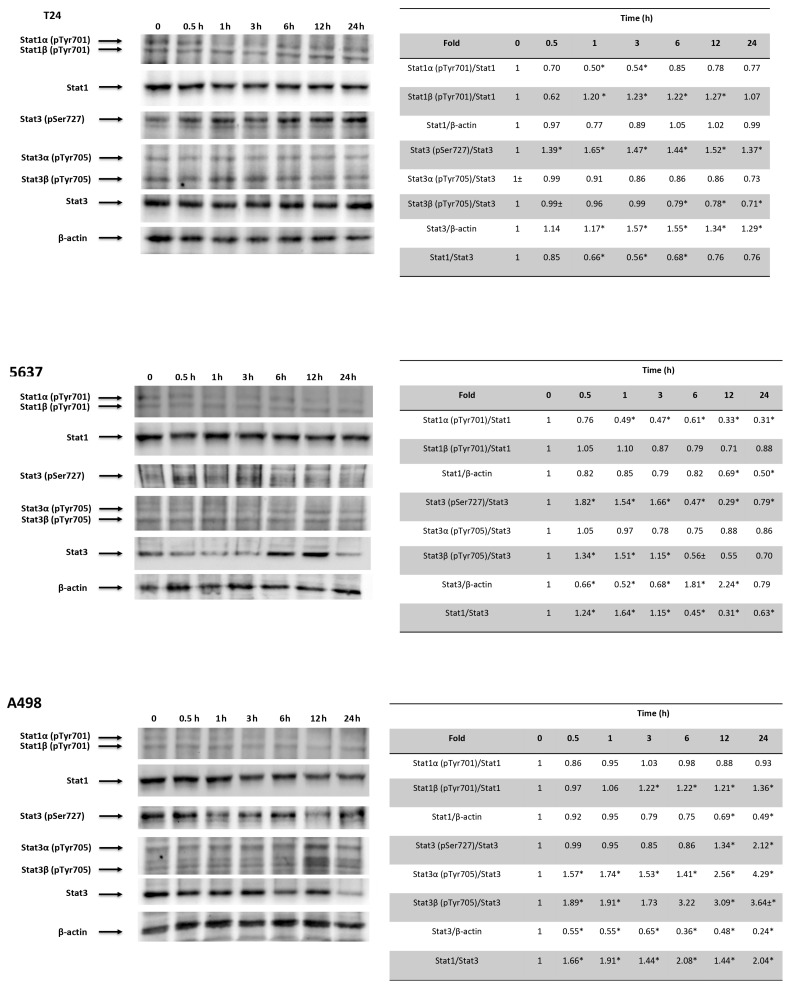
CPS effects on Stat1 and Stat3 activation. Western blot analysis of Stat1, Stat1α (pTyr701), Stat1β (pTyr701), pStat3 (pSer727), Stat3α (pTyr705), Stat3β (pTyr705) and Stat3 protein expression in T24, 5637 and A498 cell lines treated with 50 μM CPS for 12 and 24 h. Blots are representative of one of three separate experiments. Stat1α (pTyr701) and Stat1β (pTyr701) densitometry values were normalized to Stat1. Stat3 (pSer727), Stat3α (pTyr705) and Stat3β (pTyr705) densitometry values were normalized to Stat3. Stat1 and Stat3 densitometry values were normalized to β-actin, used as the loading control. Stat1 and Stat3 protein levels of treated cells were determined with respect to their levels in untreated cells. No differences were observed between untreated cells and vehicle-treated cells. Data are representative of three different experiments; * *p* < 0.01. Detailed information about the Western blotting can be found in Figure S11.

Recently, it has been demonstrated that DSB DNA damage induces activation of the Stat1 and/or Stat3 pathways [46]. Thus, the involvement of the Stat1/Stat3 signaling pathway in the CPS-induced immunomodulatory effects was evaluated by Western blot analysis at different times in CPS-treated T24, 5637 and A498 cells (Figure 8). Time-course analysis evidenced that pSer727-Stat3 levels increased, although at different times (0.5–1, 0.5–3 and 0.5–24 h) in all the CPS-treated T24, 5637 and A498 cells. Increased pTyR701-Stat1β and reduced pTyR701-Stat1α levels were evidenced in CPS-treated T24 cells. An increase in the pTyR705-Stat3β levels and a progressive reduction in the pTyR701-Stat1α levels were observed in CPS-treated 5637 cells. Finally, a progressive increase in pTyR701-Stat1β and pTyR705-Stat3βup until 24 h was evidenced in CPS-treated A498 cells. CPS increased the Stat3 protein levels in T24, whereas a reduction was evidenced in A498 cells. Moreover, in CPS-treated 5637, Stat3 levels were reduced early on and then increased later (Figure 8). Consequently, a reduction in the Stat1/3 ratio was observed in T24 cells, whereas it increased in A498 cells. Finally, in 5637 cells, the Stat1/3 ratio followed the Stat3 level trend.

## 4. Discussion

CPS exerts immunomodulatory activity in tumors, resulting in both pro-tumorigenic and anti-tumorigenic effects [7,9,10]. In this context, we found that a low dose of CPS (50 µM) modulates the expression of PD-L1 in BC and RCC lines. PD-L1 expressed in the cell membrane and cytoplasm of all cell lines analyzed increases in BC cells and decreases in A498 cells after CPS exposure. 

The 50 µM tested dose of CPS does not mediate the cytotoxic effect, but it can induce DNA damage, as evaluated by increased γ-H2AX associated with DSBs [33]. Interestingly, time-course analysis evidenced an early and weak γ-H2AX increase in T24 and 5637 cells, but strong enhancement at 12–24 h in A498 cells. It is well-demonstrated that PD-L1 expression in cancer cells is regulated in response to DSB DNA damage. This regulation requires the ATM/CHK2 and ATR/Chk1 kinases. The ATM activation promotes DSB repair as well as cell cycle checkpoint arrest, whereas ATM inhibition results in DSB repair deficiency. ATM silencing increases PD-L1 expression and sensitizes PDAC cells to immune checkpoints inhibition [47]. CHK2 activates the p53-mediated responses to DSB DNA damage. Low CHK2 expression was evidenced in locally advanced BC cells, compared to normal bladder epithelium [48]. Here, we demonstrated that inhibition of ATM/CHK2, as well as BRCA1 and TERT expression, was associated with increased PD-L1 mRNA and protein expression in p53-mutated T24 and 5637 cells. On the other hand, in p53 wild-type A498 cells, activation of a p53-dependent ATM/CHK2-mediated DNA damage response (DDR), and increased hTERT and BRCA1 transcription, are associated with PD-L1 mRNA and protein downregulation. These data were supported by Western blot analysis showing increased pSer1981-ATM, pSer19-CHK2 and Ser33/35-CHK2 phosphorylation levels only in CPS-treated A498 cells, whereas no changes in total ATM/CHK2 protein levels were found. In CPS-treated T24 and 5637 cells, we confirmed the decrease of total CHK2, and no AKT/CHK2 phosphorylation was observed. PD-L1 upregulation, after CPS-induced DNA damage to BC cells, is mediated by the canonical STING-interferon type I pathway, although other cytokines, growth factors, signal transduction, transcriptional factors and oncogenes are involved. Treatment of T24 and 5637 cells with CPS upregulates the IFNα1, IFNβ1, TGFβ1 and FGFR2 mRNA levels. A positive correlation between FGF2 and PD-1/PD-L1 immune checkpoints in BC is evidenced [49]. Moreover, in LPS-treated BC cells, there is an increase in the type I IFN pathway and PD-L1 expression [50]. In CPS-treated A498 cells, overexpression of TGFβ1/TGFβR1 and PDGFα induction are associated with PD-L1 downregulation and are accompanied by MET expression and TNFα downregulation. 

The crosstalk between activated MAPKs, STATs and NF-kB pathways seems to control the PD-L1 gene expression. The oxidative stress leading to MAPK and NF-kB activation induces PD-L1 gene transcription [51]. So, inhibition of NF-kB, Fos, Jun and Myc was evidenced in CPS-treated T24 and 5637 cells; instead, in CPS-treated A498 cells, enhanced NF-kB, E2F1 and Myc mRNA expression was evidenced. Moreover, ERBB2 expression increased in CPS-treated A498 cells, while ETS, ERBB2 and AKT levels decreased in T24 and 5637 cells. Depending on the cellular context, the c-Myc oncogene could be a positive or negative regulator of PD-L1 expression by binding to the PD-L1 promoter [52]. The homeostatic response of epithelial cells to stress activates the NF-kB pathway, leading to PD-L1 expression [53]. Further evidence also suggests that ERK-MEK signaling can regulate PD-L1 gene expression. Increased PD-L1 expression in BCs can be abrogated by MEK inhibition [54,55,56]. In agreement, MET and SYK kinases were not expressed in CPS-treated 5637 and T24 cells, while they were upregulated in A498 cells. 

CPS is the specific TRPV1 agonist [8], and the “hotness” effects reflect the activation of this receptor. TRPV1 is a non-selective cation channel sensitive to external stimuli including pH changes and also mild temperatures (41–43 °C). In accordance with the demonstration that a mild temperature upregulates the expression of PD-L1 in tumor cells [57,58], in TRPV1-positive BC cells, the TRPV1 antagonist CPZ completely reverts the CPS-induced increase in PD-L1, supporting the role of TRPV1 in PD-L1 regulation. 

CPS exerts both oxidative [59] and anti-oxidative [60] properties. Oxidative stress can impact PD-L1 expression in cancer cells. A complex interplay between ROS drivers and PD-L1 expression reveals that, depending on target cells and involved pathways, ROS generation can result in both up- and downregulation of PD-L1 expression. Bailly et al. first described the effects of a ROS-modulating drug on PD-L1 expression [38]. Here, ROS generation was evidenced after CPS treatment in A498 cells, but not in 5637 and T24 cells. Moreover, the ROS inhibitor, NAC, completely reverted the reduction in PD-L1 expression in A498 cells. The absence of ROS in CPS-treated BC cells may be the result of the reduced sensitivity of the DCFDA to detect ROS. In that context, we performed the Oxy-Blot assay, which has a high sensitivity to carbonyl residues. Data showed increased levels of oxidized proteins at 1–3 h after CPS treatment in T24 and 5637 cells. We also demonstrated that NAC, as an inhibitor of protein carbonylation, completely reverts the CPS-mediated PD-L1 increase in BC cells. The presence of carbonyl groups indicated protein peroxidation and cellular carbonyl stress, resulting in protein damage. Recent data [42] suggest the participation of oxidative stress in BC development, and a positive correlation between BC progression and higher levels of carbonyl proteins and lipid peroxidation in BC patients. The cellular redox homeostasis is finetuned by a balance between antioxidant and pro-oxidant molecules. In this regard, CPS causes ROS generation, which disrupts redox homeostasis. This effect can arise not only as a direct effect of oxidant molecules but also by inhibition of the antioxidant activity [38]. In this regard, the NME4 belonging to the nucleoside diphosphate kinase (NDPK)/Nm23 family functions to reduce the ROS-induced genome instability. NME4 catalyzes reactions that transfer the terminal phosphatase of a nucleotide triphosphate to a nucleoside diphosphate to equilibrate the NDP and NTP pools in cells [61,62]. Silencing of NME4 increases the oxidative stress at cellular levels, and delaying DSB repair causes ROS-mediated genome instability [63]. In regard to CPS treatment, a reduction of NME4 protein expression was detected in the 5637 and T24 cells at 12–24 h after treatment. Thus, in CPS-treated BC cells, the reduction of the anti-oxidative NME4 protein likely promotes DSB DNA damage, a defective DDR and genome instability. 

DSB activates Stat1/Stat3 signaling and is required for DSB-dependent modulation of PD-L1 expression. Stat3 becomes phosphorylated at Tyr705 and Ser727 on stimulation. Phospho-Tyr705 (pY705) stabilizes the Stat3 dimer with reciprocal interactions between pY705 and the SH2 of the other molecule, and phospho-Ser727 (pS727) accelerates pY705 dephosphorylation [64]. ROS influence Stat3 signaling by inhibiting phosphatases and activating kinases, and Stat3 inhibition is accompanied by increased ROS levels [65]. The Stat1 signaling pathway can be activated by TNFα and TGFβ through TyR701 phosphorylation. TGFβ1 activates the Stat1 pathway by Stat1α Ser727 phosphorylation [66]. In regard to Stat3 activation, the TRPV1 agonist resiniferatoxin induces Stat3 phosphorylation; moreover, Stat3 activation was found to be lower in Trpv1^−/−^ compared to Trpv1^+/+^ mice [67]. Stat3 can act both as a potent tumor promoter and a tumor suppressor factor [68]. In particular, Stat3α, the full-length version of Stat3, regulates the oncogenic functions of Stat3. Conversely, Stat3β, generated by alternative splicing and lacking the C-terminal transactivation domain, inhibits cancer progression, acting as a repressor of Stat3. Finally, deactivation of Stat3 by chemical inhibitors or genetic silencing results in decreased PD-L1 expression [69].

Here, we evidenced that CPS-induced TRPV1-mediated DSB DNA damage is associated with an increase in Stat3 (pSer727) in CPS-treated T24 and 5637 cells. pTyR701-Stat1β and reduced pTyR701-Stat1α levels were evidenced in CPS-treated T24 cells. An increase in the pTyR705-Stat3β levels and a progressive reduction of the pTyR701-Stat1α levels were observed in CPS-treated 5637 cells. Finally, a progressive increase of pTyR701-Stat1β and pTyR705-Stat3β up to 24 h was evidenced in CPS-treated A498 cells. However, activation of Stat1 or Stat3 is not sufficient to predict Stat effects. Indeed, the phosphorylation levels may also depend on the ratio between Stat1 and Stat3 proteins, with Stat1 counteracting the pro-tumorigenic Stat3 signaling [70,71]. In BCs, Stat3 is implicated in the progression from carcinoma in situ to invasive BCs. Stat3 signaling acts downstream of inflammatory cytokines released during bladder tumorigenesis; Stat3 activation is directly related to the malignant behavior of T24 BC cells, and inhibition of pStat3 levels reduces T24 cell invasion [72]. Moreover, Stat3 is also involved in RCC carcinogenesis [72]. In this scenario, CPS-induced PD-L1 reduction is associated with a Stat1^high^Stat3^low^ phenotype in A498 cells and a Stat1^low^Stat3^high^ phenotype in CPS-treated T24 cells, whereas an early decrease in the Stat1/3 ratio, followed by a delayed increase, was observed in CPS-treated 5637 cells. Finally, mounting evidence suggests that high hTERT activity is tightly associated with Stat3 activation, cancer progression and poor outcomes, and Stat3 knock-out downregulates hTERT expression [73]. 

Previous reports evidenced a correlation between tumor PD-L1 expression and more aggressive tumor behavior. This result is related to the role of the PD-1/PD-L1 pathway [27]. Indeed, multiple solid tumor types, including RCC [74] and BC [32], generate an immunosuppressive tumor microenvironment by expressing PD-L1, thereby avoiding T cell-mediated cytotoxicity, correlated with a worse prognosis. In BC and RCC, high tumoral PD-L1 levels are considered a negative prognostic factor [75]. Furthermore, we recently reported a correlation between high PD-L1 expression and shorter recurrence-free survival in circulating tumor cells isolated from peripheral blood in NMIBC [76]. 

**Figure 9:**
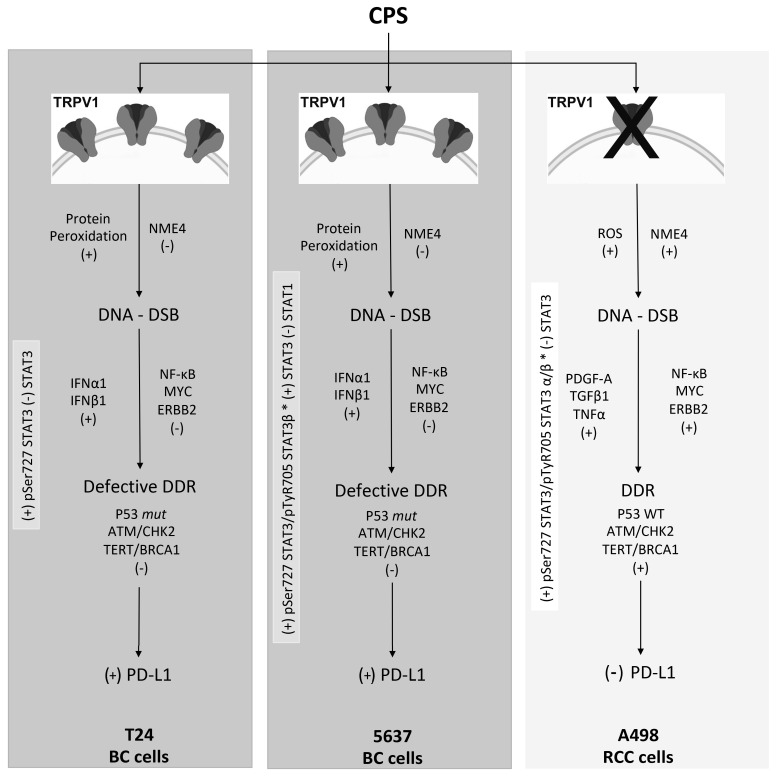
Molecular mechanisms of CPS-induced PD-L1 modulation in BC and RCC lines. ROS, reactive oxygen species; DDR, DNA damage response; DSB, double-strand breaks; *mut*, mutant; WT, wild-type; +, increased/activated; -, decreased/deactivated.

Overall, our data evidenced the two faces of the CPS-mediated immunomodulatory effect in BC and RCC cell lines (Figure 9). In 5637 and T24 cells, CPS promotes, in a TRPV1-dependent manner, upregulation of PD-L1 expression. On the contrary, in A498 cells, CPS acts in a TRPV1-independent manner by inducing PD-L1 downregulation. As such, the CPS-induced increase in PD-L1 can promote BC progression, but on the other hand, it may also represent a potential target for immunotherapy with anti-PD-1/PD-L1 antibodies. However, although at present, some clinical data support a good effect of the anti-PD-L1 immunotherapy, alone or in combination with BCG, in BC patients [77,78,79], no conclusive evidence of the predictive role of cancer’s PD-L1 levels in the anti-PD-1/PD-L1 immunotherapy outcome have been reported yet [32,80]. In RCC, the reduction of PD-L1 expression with CPS treatment could allow for a better anti-tumor immune response, so CPS or TRPV1 agonists could be suggested as immuno-adjuvants in the RCC therapy. 

## 5. Conclusions

Knowledge of the different signaling pathways and molecular drivers triggered by CPS treatment might permit us to identify different targets and levels of immunoregulation, to better evaluate the therapeutic strategies and the potential use of immunoadjuvants for BC or RCC patients.

## Figures and Tables

**Table 1:** Transcriptional factors, oncogenes and signaling molecule gene expression in CPS (50 μM)-treated 5637, T24 and A498 cells.

Unigene	GenBank ID	Symbol	Cell Lines					
			5637		T24		A498	
			4 h	12 h	4 h	12 h	4 h	12 h
NM_003998	Nuclear factor of kappa light polypeptide gene enhancer in B cells-1	NF-kB1	1.4	−1.2	1.1	−1.8	2.0 *	−1.2
NM_020529	Nuclear factor of kappa light polypeptide gene enhancer in B cells inhibitor alpha	NF-kBIA	−2.3 *	−2.7 *	−2.1 *	−1.7	−1.2	−1.7
NM_005225	E2F transcriptional factor 1	E2F1	N/A	N/A	N/A	N/A	IND *	3.2 *
NM_005239	V-ETs erythroblastosis virus E26 oncogene homolog 2	ETS2	−1.1	−2.2 *	−1.0	−2.8 *	1.7	−1.2
NM_005252	V-Fos FBJ murine osteosarcoma viral oncogene homolog	FOS	−1.4	−3.0 *	−1.9	−2.0 *	−2.3*	−7.7 *
NM_002228	V-Jun sarcoma virus 17 oncogene homolog	JUN	−1.3	−2.4 *	−1.1	−2.8 *	−2.6*	−3.1 *
NM_002467	V-mycmyelocitomatosis viral oncogene	MYC	1.3	−1.5	1.5	−1.9	5.0 *	2.0 *
NM−005163	V-Akt murine thymoma viral oncogene homolog 1	AKT	SIL *	−2.9 *	−2.3 *	−3.4 *	N/A	N/A
NM_004448	V-Erb-b2 erythroblastic leukemia viral oncogene homolog 2	ERBB2	1.1	−2.4 *	1.1	−2.4 *	3.2 *	1.5
NM_002880	V-Raf-1 murine leukemia viral oncogene homolog 1	RAF1	1.0	−1.2	1.1	−1.0	1.5	1.2
NM_000245	Met proto-oncogene	MET	N/A	N/A	N/A	N/A	IND *	1.9
NM-002755	Mitogen-activated protein kinase kinase 1	MAPKK1	−2.6 *	−1.4	−2.4 *	−1.0	2.7 *	2.0 *
NM_181504	Phosphoinositide-3-kinase, regulatory subunit polypeptide 1	PI3K	1.6	1.1	1.8	1.6	1.0	−1.1
NM_003177	Spleen tyrosine kinase	SYK	N/A	N/A	N/A	N/A	IND *	2.8 *

Fold expression < or > 2; * *p* < 0.01 vs. vehicle-treated cells; h = hours; (−) decreased; IND = induced; SIL = silenced; N/A = not detected.

**Table 2:** Cytokines, growth factors and receptors’ gene expression in CPS (50 μM)-treated 5637, T24 and A498 cells.

Unigene	GenBank ID	Symbol	Cell Lines					
			5637		T24		A498	
			4 h	12 h	4 h	12 h	4 h	12 h
NM_024013	Interferon alpha 1	IFN-α1	17.4 *	12.2 *	6.2 *	4.9 *	1.5	1.6
NM_002176	Interferon beta 1 fibroblast	IFN-β1	24.5 *	19.8 *	9.5 *	5.3 *	1.4	1.3
NM_000618	Insulin-like growth factor 1	IGF-1	N/A	N/A	N/A	N/A	N/A	N/A
NM_000584	Interleukin-8	IL-8	1.3	1.1	1.1	−9.1 *	1.8	1.1
NM_002607	Platelet-derived growth factor alpha polypeptide	PDGF-A	N/A	N/A	N/A	N/A	IND *	3.0 *
NM_002607	Platelet-derived growth factor beta polypeptide	PDGF-B	−1.1	−1.5	−1.7	−1.9	−3.7 *	−3.9 *
NM_000660	Transforming growth factor beta-1	TGF-β1	1.1	1.0	1.2	2.5 *	1.5	4.0 *
NM_000594	Tumor necrosis factor alpha	TNF-α	1.9	1.7	1.4	−1.6	2.9 *	2.0 *
NM_000141	Fibroblast growth factor receptor 2	FGFR2	2.3 *	−1.3	2.3 *	−1.3	1.5	1.1
NM_003246	Transforming growth factor beta receptor 1	TGFβR1	1.0	1.2	1.6	2.1 *	2.0 *	2.2 *
NM_003862	Tumor necrosis factor receptor superfamily member 10b	TNFRSM10b	−1.4	−2.4 *	−1.4	−2.4 *	2.8 *	1.6
NM_001065	Tumor necrosis factor receptor superfamily member 1A	TNFRSM1A	−2.0 *	−3.6 *	−2.0 *	−3.6 *	2.0 *	1.1
NM_003790	Tumor necrosis factor receptor superfamily member 25	TNFRSM25	1.7	−1.6	1.7	−1.6	2.8 *	2.0 *

Fold expression < or >2; * *p* < 0.01 vs. vehicle-treated cells; h = hours; (−) decreased; IND = induced; N/A = not detected.

## Data Availability

The data that support the findings of this study are available from the corresponding author on request.

## Supplementary Materials

The following supporting information can be downloaded at: https://www.mdpi.com/article/10.3390/cancers14112644/s1, Figure S1: CPS does not induce apoptotic cell death; Figure S2: TRPV1 silencing in 5637 and T24 cell lines, Figure S3: CPS does not influence ROS production; Figure S4: CPS does not influence mitochondrial transmembrane potential, Figure S5: Full Western blot for Figure 1, Figure S6: Full Western blot for Figure 2, Figure S7: Full Western blot for Figure 4, Figure S8: Full Western blot for Figure 5, Figure S9: Full Western blot for Figure 6, Figure S10: Full Western blot for Figure 7, Figure S11: Full Western blot for Figure 8.
