# Supplementary material for: Unveiling the Molecular Mechanisms Driving the Capsaicin-Induced Immunomodulatory Effects on PD-L1 Expression in Bladder and Renal Cancer Cell Lines
Source: Cancers (Basel). 2022 May 26;14(11):2644. doi: 10.3390/cancers14112644 (PMC9179445; doi:10.3390/cancers14112644)
Supplement: Supplementary file 1 [file cancers-14-02644-s001.zip › cancers-1691984-supplementary.pdf]

Supplementary Materials

Unveiling the Molecular Mechanisms Driving the Capsaicin-Induced Immunomodulatory Effects on PD-L1 Expression in Bladder and Renal Cancer Cell Lines

Maria Beatrice Morelli, Oliviero Marinelli, Cristina Aguzzi, Laura Zeppa, Massimo Nabissi, Consuelo Amantini, Daniele Tomassoni, Federica Maggi, Matteo Santoni and Giorgio Santoni

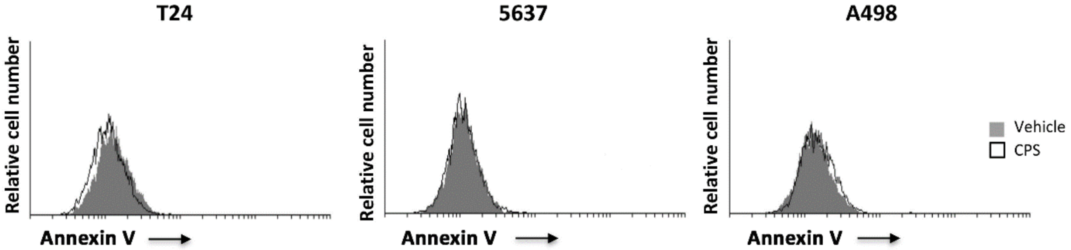

Figure S1. CPS does not induce apoptotic cell death.

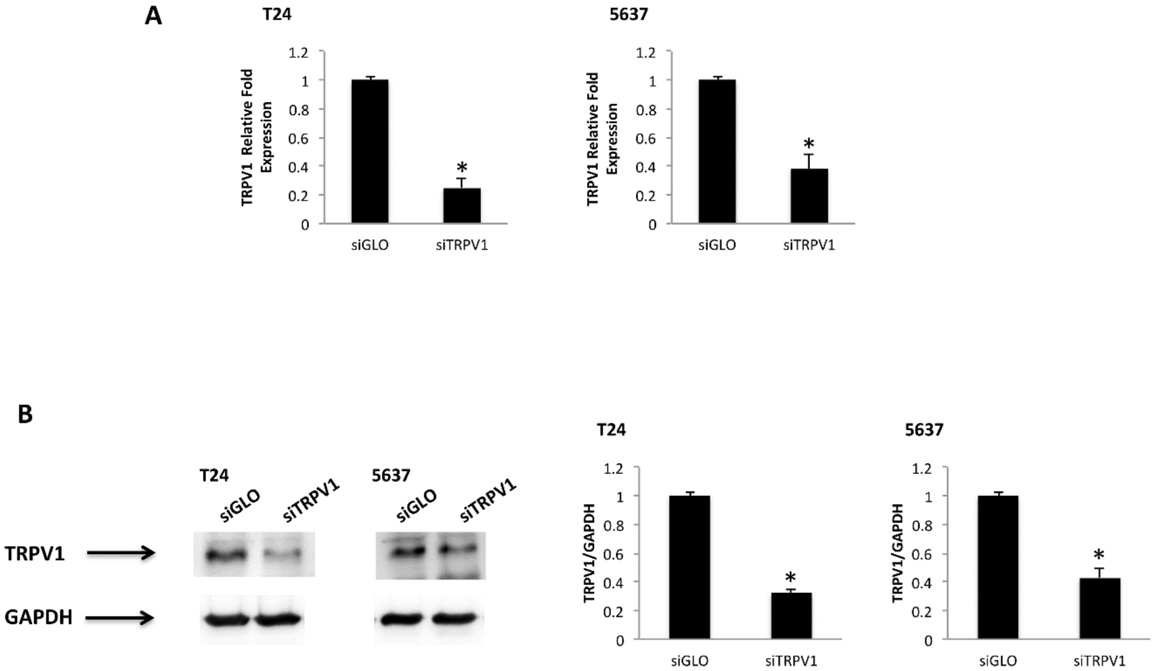

Figure S2. TRPV1 silencing in 5637 and T24 cell lines.

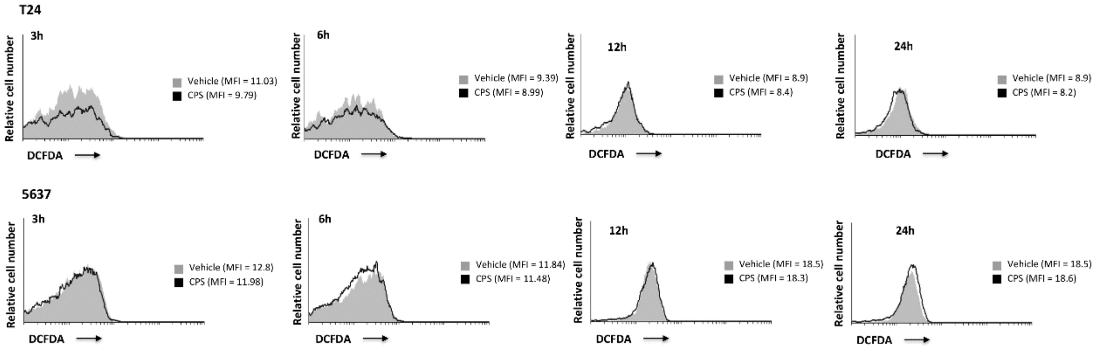

Figure S3. CPS does not influence ROS production.

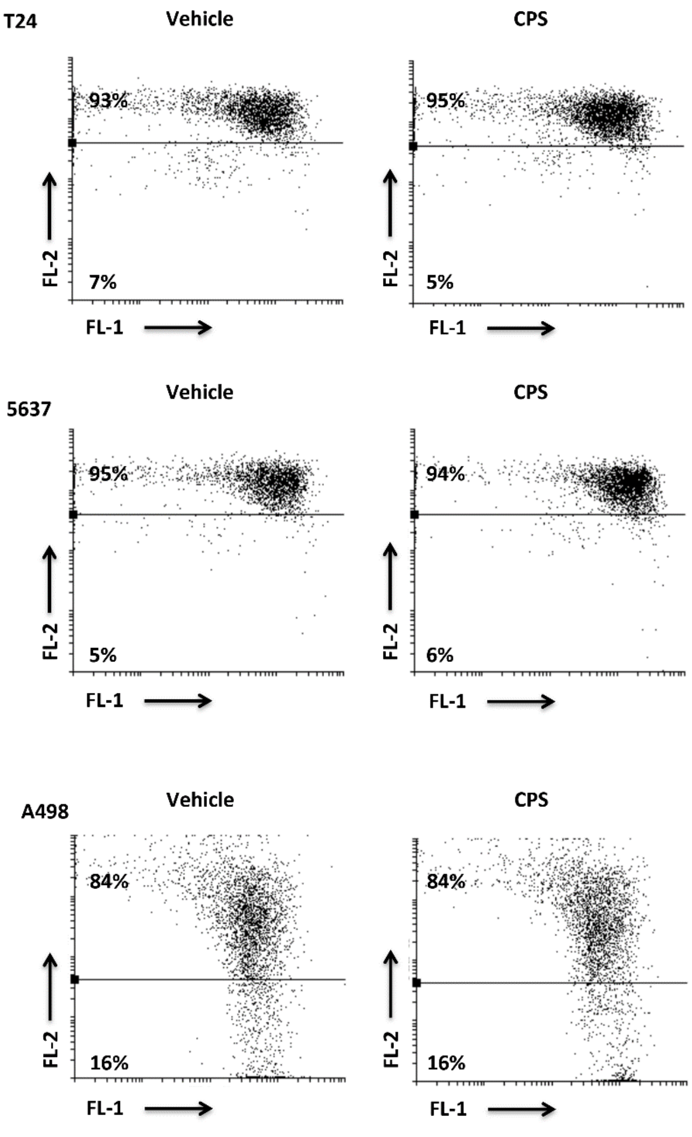

Figure S4. CPS does not influence mitochondrial transmembrane potential.

Figure 1

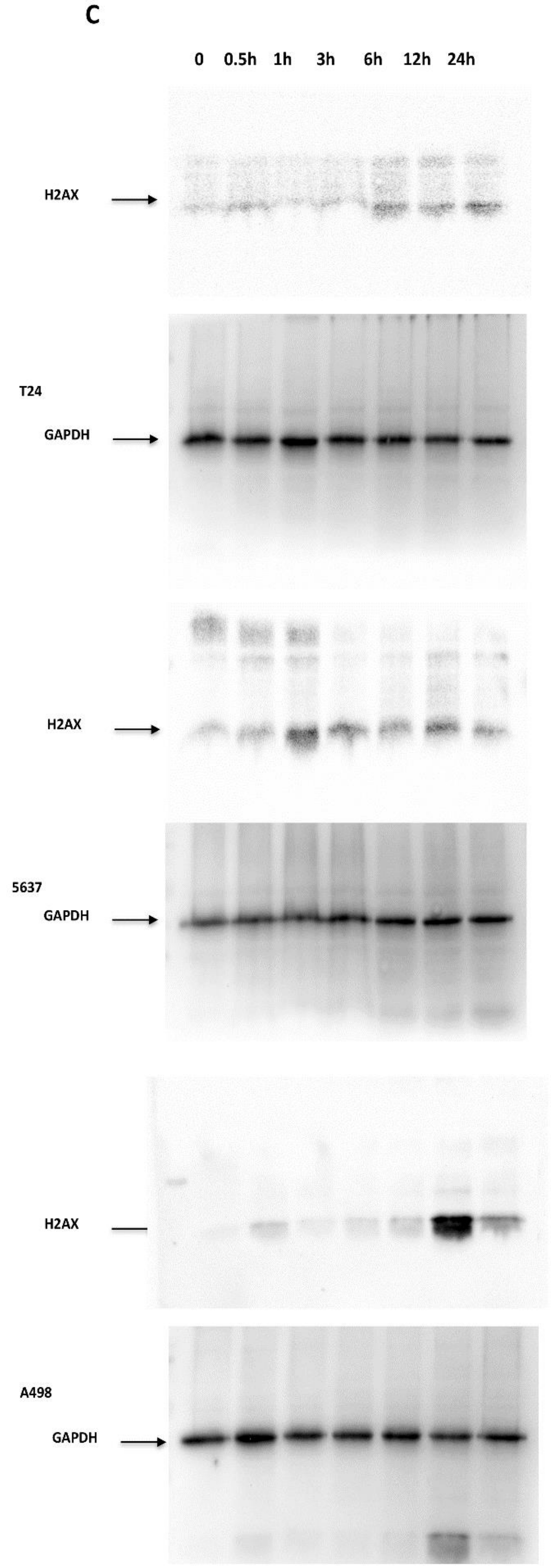

Figure S5. Full Western blot for Figure 1.

Figure 2

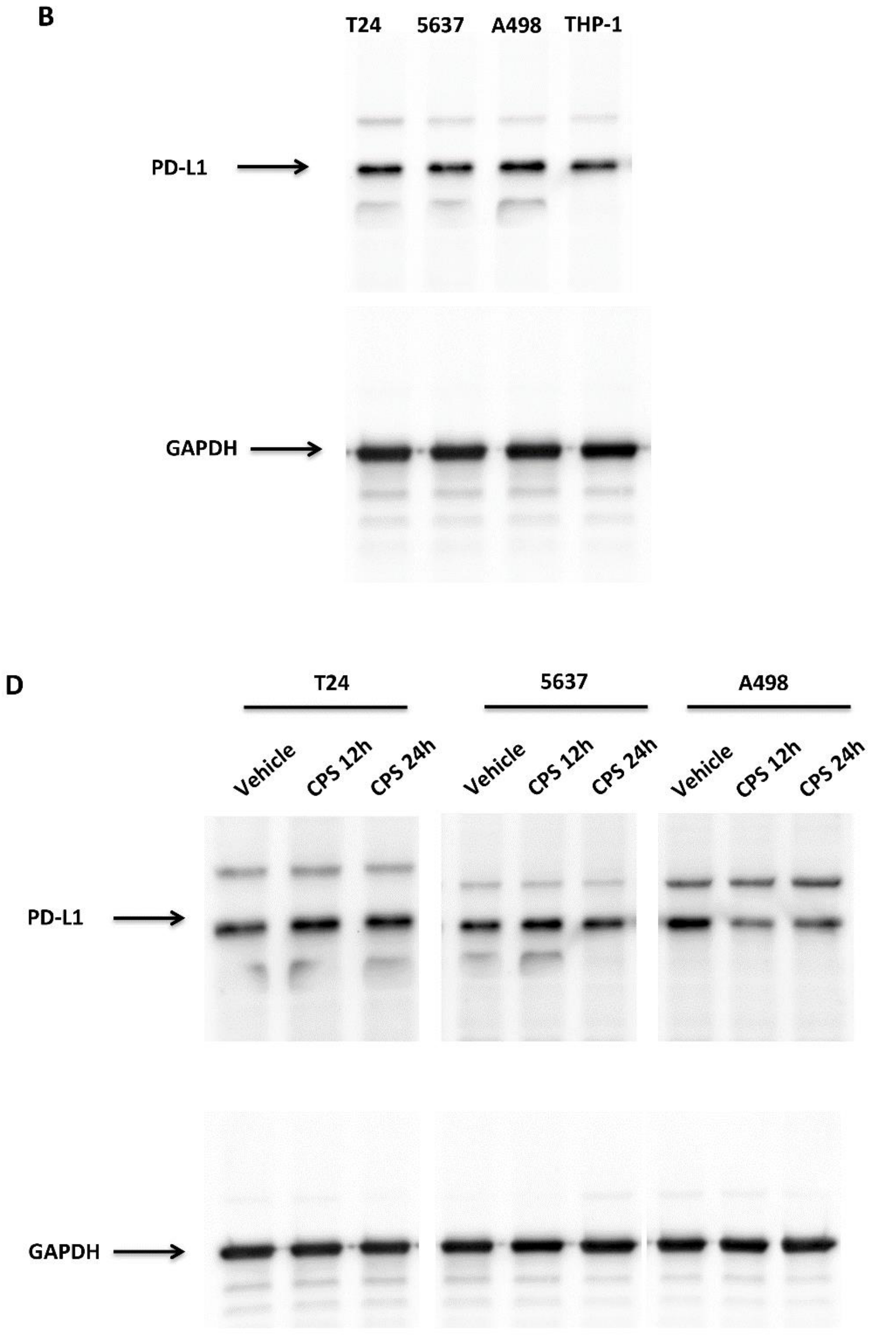

Figure S6. Full Western blot for Figure 2.

Figure 4

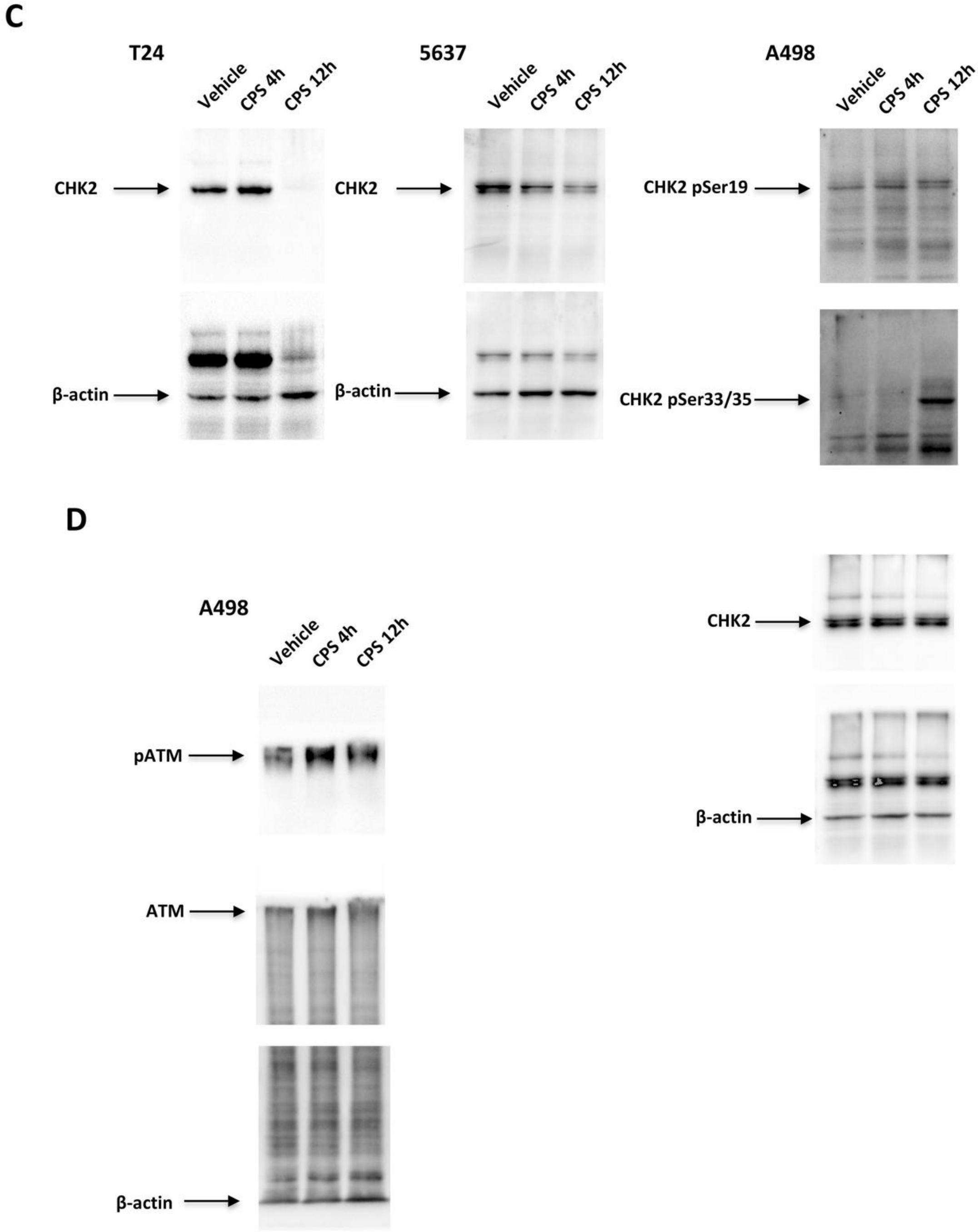

Figure S7. Full Western blot for Figure 4.

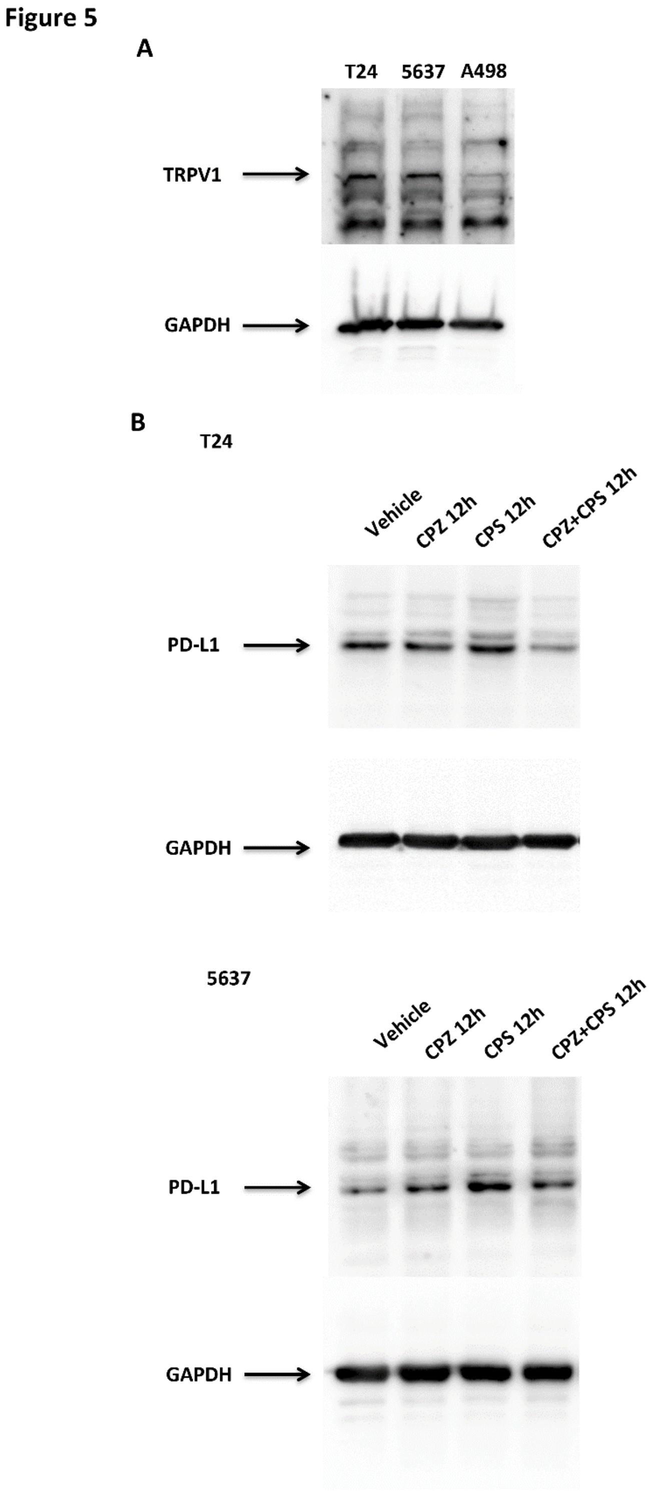

Figure S8. Full Western blot for Figure 5.

Figure 6

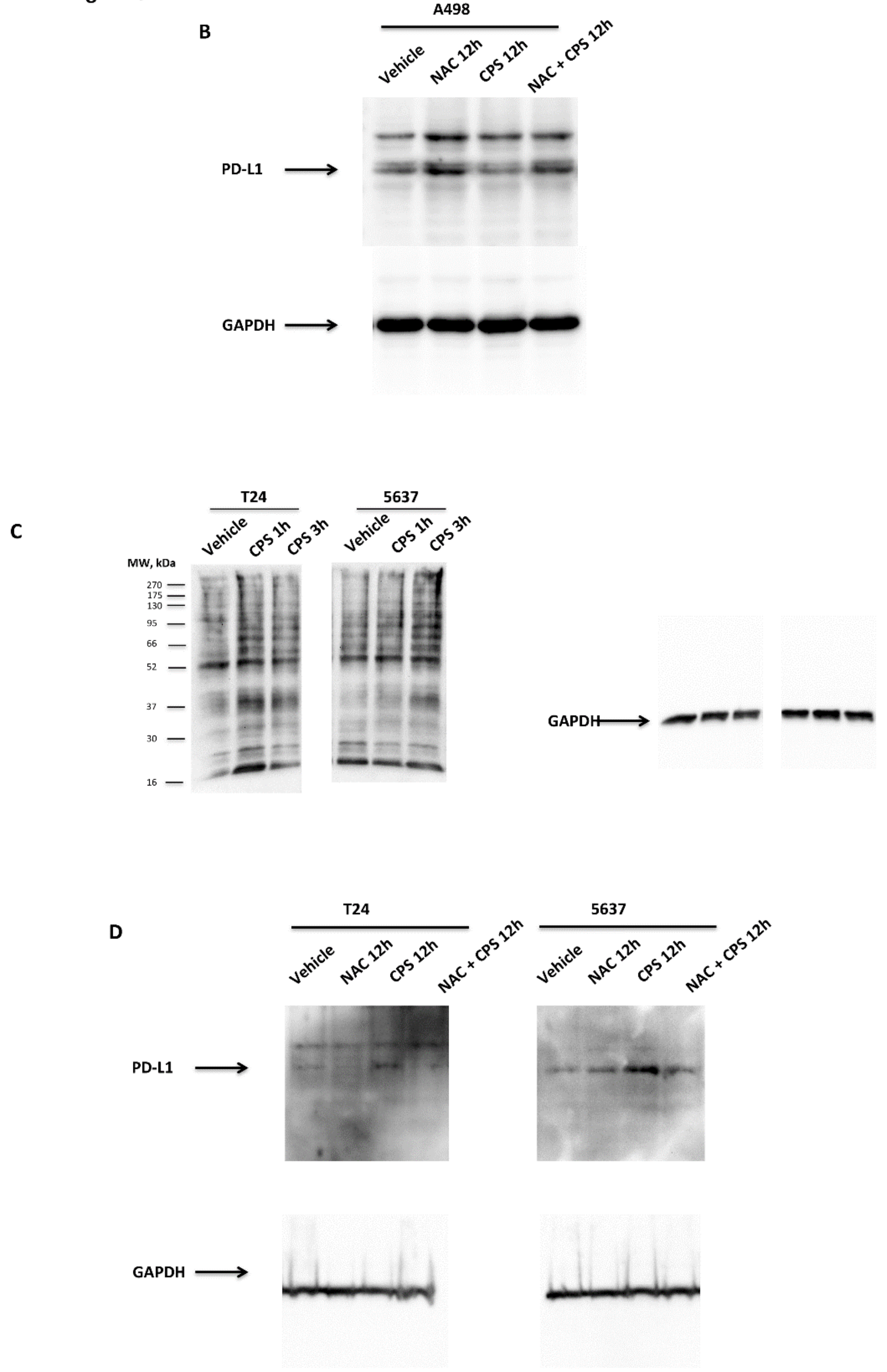

Figure S9. Full Western blot for Figure 6.

Figure 7

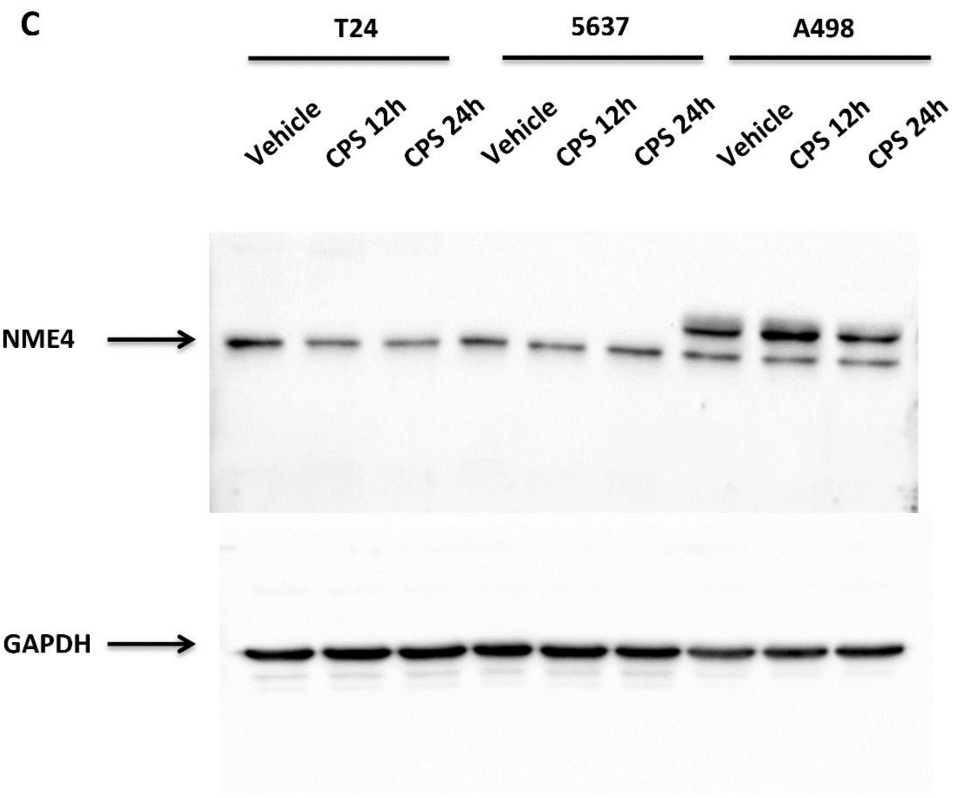

Figure S10. Full Western blot for Figure 7.

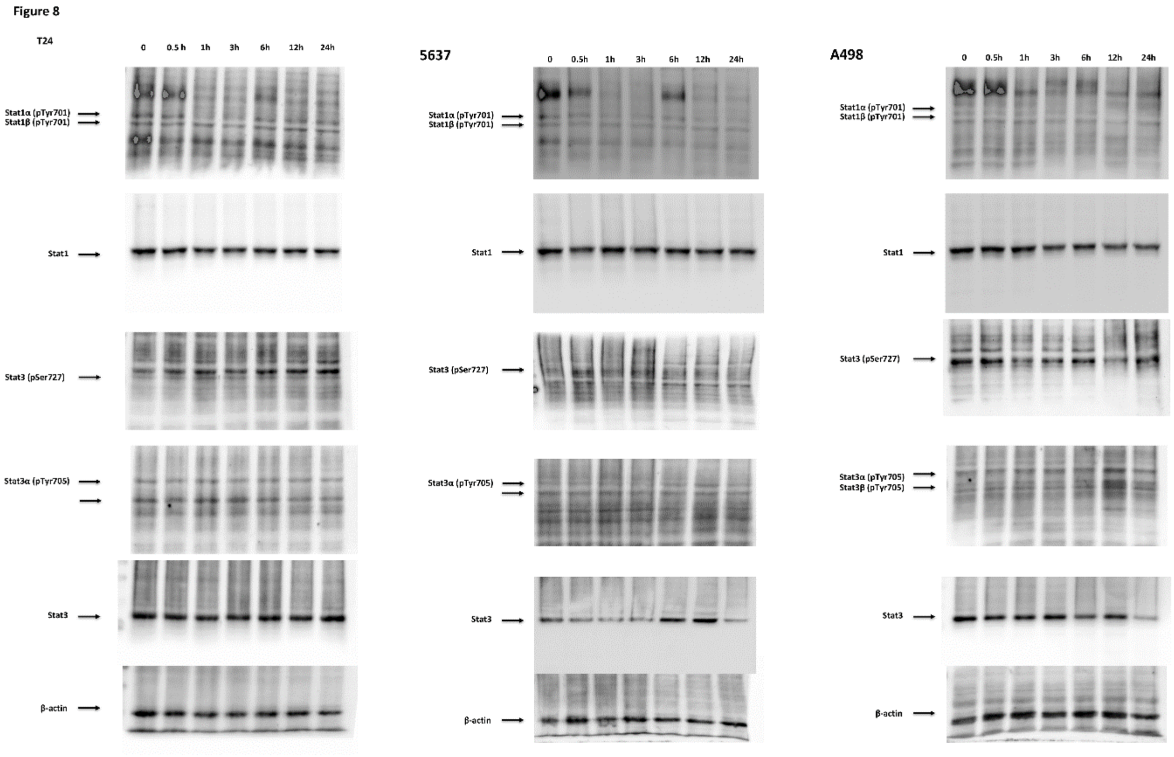

Figure S11. Full Western blot for Figure 8.
